# Establishment and characterization of immortalized human breast cancer cell lines from breast cancer patient-derived xenografts (PDX)

**DOI:** 10.1038/s41523-021-00285-x

**Published:** 2021-06-18

**Authors:** Yongxian Zhuang, Jordan M. Grainger, Peter T. Vedell, Jia Yu, Ann M. Moyer, Huanyao Gao, Xiao-Yang Fan, Sisi Qin, Duan Liu, Krishna R. Kalari, Matthew P. Goetz, Judy C. Boughey, Richard M. Weinshilboum, Liewei Wang

**Affiliations:** 1 Division of Clinical Pharmacology, Department of Molecular Pharmacology and Experimental Therapeutics, Mayo Clinic, Rochester, MN USA; 2 Division of Biomedical Statistics and Informatics, Department of Health Sciences, Mayo Clinic, Rochester, MN USA; 3 Department of Lab Medicine and Pathology, Mayo Clinic, Rochester, MN USA; 4 Department of Health Sciences Research, Mayo Clinic, Rochester, MN USA; 5 Department of Oncology, Mayo Clinic, Rochester, MN USA; 6 Department of Surgery, Mayo Clinic, Rochester, MN USA

**Keywords:** Cancer models, Breast cancer, Experimental models of disease, Cancer models, Cancer genomics

## Abstract

The application of patient-derived xenografts (PDX) in drug screening and testing is a costly and time-consuming endeavor. While cell lines permit extensive mechanistic studies, many human breast cancer cell lines lack patient characteristics and clinical treatment information. Establishing cell lines that retain patient’s genetic and drug response information would enable greater drug screening and mechanistic studies. Therefore, we utilized breast cancer PDX from the Mayo Breast Cancer Genome Guided Therapy Study (BEAUTY) to establish two immortalized, genomically unique breast cancer cell lines. Through extensive genetic and therapeutic testing, the cell lines were found to retain the same clinical subtype, major somatic alterations, and drug response phenotypes as their corresponding PDX and patient tumor. Our findings demonstrate PDX can be utilized to develop immortalized breast cancer cell lines and provide a valuable tool for understanding the molecular mechanism of drug resistance and exploring novel treatment strategies.

## Introduction

Immortalized cell lines have been widely used for studying cancer biology and treatment response^1^. The major advantages of using cultured cell lines are their prolonged conservation of genetic and molecular features and can be easily managed and maintained using standard culture conditions to provide enough material for cancer research in a short time period^2^. However, most commercially available cell lines were established decades ago, with the first human breast cancer cell line created in 1958^3^, and have limited patient-related clinical and treatment information. Establishing cell lines that retain this information will better assist researchers in understanding the molecular biology of cancer.

Ex vivo culture of fresh patient tumor tissue or establishing patient-derived xenografts (PDX) in mice, provide models closely representing the heterogeneity and drug response of patient tumors^4^. We previously established multiple breast cancer PDX models from primary breast cancer patient tumors recruited in a prospective trial, BEAUTY^5,6^. Utilizing these PDX models has enabled us to identify novel pharmacogenomic biomarkers associated with various therapies^6,7^. Furthermore, we have successfully generated PDX-derived organoids by dissociating PDX tumors into single-cell suspensions and culturing them in vitro and demonstrated their importance in understanding cancer pathology and drug resistance mechanisms^7^. However, the costs associated with the maintenance and long turnaround time to generate PDX limit their widespread use in laboratory practice. On the other hand, the establishment of immortalized primary breast cancer cell lines from PDX tumors could provide valuable tools for mechanistic studies and elucidating alternative therapies in a relatively cost-effective way.

The process to acquire immortalized cancer cell lines is complex with unpredictable results^8,9^. The ability of cancer cells to grow on plastic tissue culture plates varies based on the cancer cell histology, tumor grade, presence of specific genetic aberrations as well as appropriate nutritional support^4^. The human breast cancer cell lines extensively studied by investigators (MCF-7, T47D, MDA-MB-231, and SKBR-3) were established from metastatic lesions, making them less likely to accurately recapitulate the genetic composition or biological behavior of primary breast tumors^10^. While others have successfully established cell lines from tumors^11,12^, only biopsies were obtained from BEAUTY patients, limiting tumor availability for in vitro sub-culture. Previously, Cavotelli et al. and Matossian et al. demonstrated the advantage of utilizing in vivo systems to generate immortalized cell lines^13–15^. They suggested sub-culturing tumor biopsies in vivo not only expands the number of tumor cells but enables tumors to adapt to their surrounding microenvironment, ensuring proliferative growth and easy passage-traits essential to the immortalization of the tumor cells^15^. As a result, when PDX tumors were plated in a single-cell suspension in cell culture, immortalized cell lines were established. These examples provide us with the rationale to examine whether it is possible to derive immortalized cell lines from our primary breast cancer PDX tumors.

Here we demonstrate not only the feasibility of establishing unique HER2+ and triple-negative immortalized primary breast cancer cell lines from their respective PDX tumors, but also draw genetic and drug phenotype comparisons between their respective organoid, PDX, and original patient primary tumor. We hope that our findings will provide researchers with additional knowledge and tools to better elucidate the mechanisms behind drug resistance and identify novel therapies for breast cancer patients in the era of precision medicine.

## Results

### Established cell lines are unique breast cancer cell lines

**Fig. 1:**
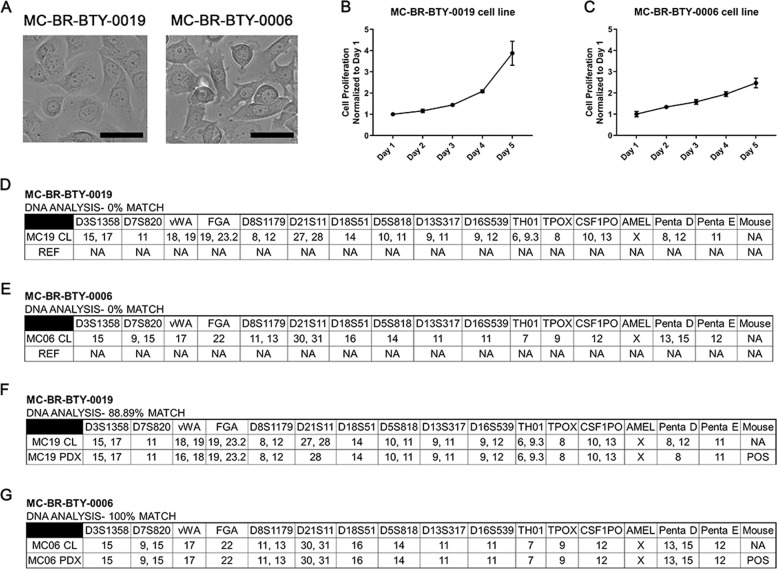
Established cell lines are unique breast cancer cell lines. **A** The images of MC-BR-BTY-0019 and MC-BR-BTY-0006 were presented after they were established as immortalized cell lines. Images were taken at ×20; Scale bar represents 20 μm. **B**, **C** 30,000 cells and 85,000 cells were plated onto 24-well plates for the proliferation assay for MC-BR-BTY-0019 and MC-BR-BTY-0006, respectively. The proliferation curves are normalized to day 1. Error bars represent SEM. DNA analysis of **D** MC-BR-BTY-0019 cell line, **E** MC-BR-BTY-0006 cell line, **F** MC-BR-BTY-0019 PDX, and **G** MC-BR-BTY-0006 PDX was demonstrated by STR profiling. REF refers to allele comparisons to all cell lines available in ATCC, DSMZ, JCRB STR profile databases. “NA” designates no similar allele calls compared to the reference.

Immortalized cell lines, MC-BR-BTY-0019 and MC-BR-BTY-0006, were established from HER2+ and triple-negative primary breast cancer PDX models maintained in NSG mice, respectively. Cells were isolated, cultured, and trypsin-passaged in optimized culture medium and conditions as described in the “Methods” section for ~2 months until being able to be passaged continuously without an apparent sign of senescence; at which point the cells were considered to be immortalized. MC-BR-BTY-0019 cells were round-spindle shape and grew as a monolayer with tight adherence to the plate, while MC-BR-BTY-0006 cells were round shape and had loose adherence to the plates (Fig. 1A). MC-BR-BTY-0019 initially grows slowly after plating, then faster after adhering for 2 days (Fig. 1B). The doubling time for MC-BR-BTY-0019 and BR-BTY-0006 was about 24–48 and 48–72 h, respectively (Fig. 1B, C).

Next, we performed short tandem repeat (STR) profiling to confirm the unique genetic identity of our cell lines. Based on testing results obtained from the analysis of 15 autosomal STR loci and the gender identity locus amelogenin, the profile of MC-BR-BTY-0019 and MC-BR-BTY-0006 did not match any existing cell lines documented by the ATCC, DSMZ, and JCRB human cell line STR profile databases (Fig. 1D, E); suggesting MC-BR-BTY-0019 and MC-BR-BTY-0006 had unique profiles. Furthermore, we performed STR profile comparisons with their PDX of origin (Fig. 1F, G). MC-BR-BTY-0019 had an 88.89% identity match and MC-BR-BTY-0006 had a 100% identity match with their respective PDX tissues. An identity match score above 80% indicates that the cell line is consistent with its PDX of origin. Since these two cell lines were isolated from PDX in immune-deficient mice, mouse DNA detection was also included in the STR profiling. No mouse DNA was detected from these two established cell lines as shown by the electropherograms of analyzed data (Supplemental Fig. 2B). We maintained the cell lines by passing them two to three times every week for 6 months while retaining the same STR profile (Supplemental Fig. 5).

### Two newly established breast cancer cells lines present significantly abnormal karyotypes

**Fig. 2:**
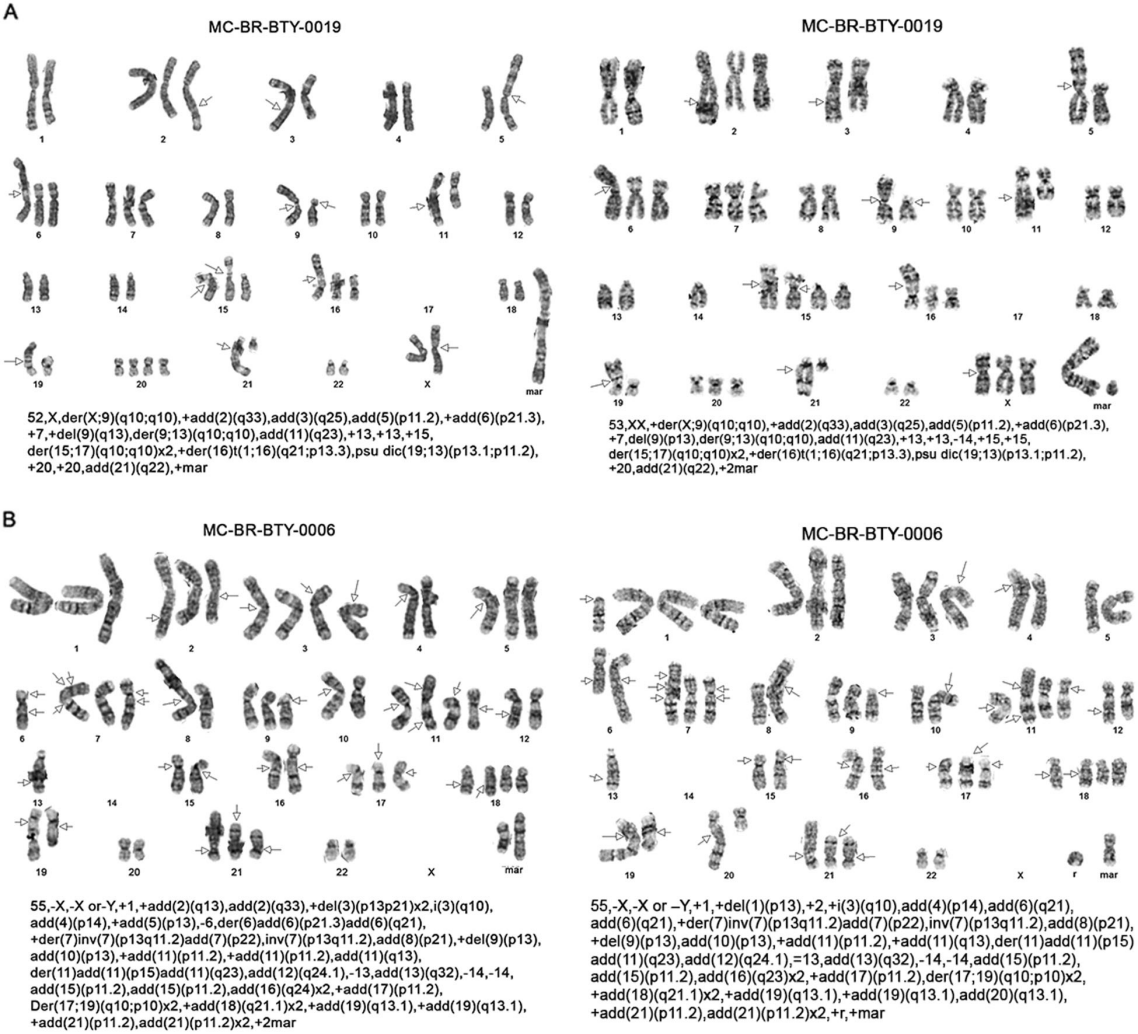
Two newly established breast cancer cells lines presented massive abnormal karyotypes. **A**, **B** Cytogenetics G-band staining of chromosomes is presented and chromosome alteration is described for two cells, MC-BR-BTY-0019 and MC-BR-BTY-0006 cell lines, respectively. Each image is a karyotype of one cell. Text below each karyotype refers to International System for the Human Cytogenetic Nomenclature (ISCN) of the karyotype to address the number of chromosomes observed, the sex chromosome, and the abnormalities observed per chromosome. The arrows indicate breakpoints or abnormalities.

Karyotype images of the two cells, MC-BR-BTY-0019 and MC-BR-BTY-0006 are shown in Fig. 2A, B, respectively. MC-BR-BTY-0019 exhibited a very complex hyper-diploid karyotype, which included numerous clonal structural rearrangements including an unbalanced rearrangement of chromosome 17 resulting in loss of 17p, several unbalanced whole-arm rearrangements, and a very large chromosome area composed of several chromosome regions derived from translocation, among numerous other abnormalities. Eleven of these metaphases represent a tetraploid subclone (Fig. 2A).

Similar to MC-BR-BTY-0019, MC-BR-BTY-0006 exhibited a very complex hyper-diploid karyotype which included numerous clonal structural rearrangements including an isochromosome 3q, a doubled version of an unbalanced, whole arm rearrangement involving chromosomes 17 and 19, resulting in loss of 17p and doubled versions of structural abnormalities of 18q and 21p, as well as numerous other unbalanced abnormalities (Fig. 2B).

### MC-BR-BTY-0019 and MC-BR-BTY-0006 maintained the molecular subtype characteristics and drug sensitivity of their corresponding PDX-derived organoids in vitro

**Fig. 3:**
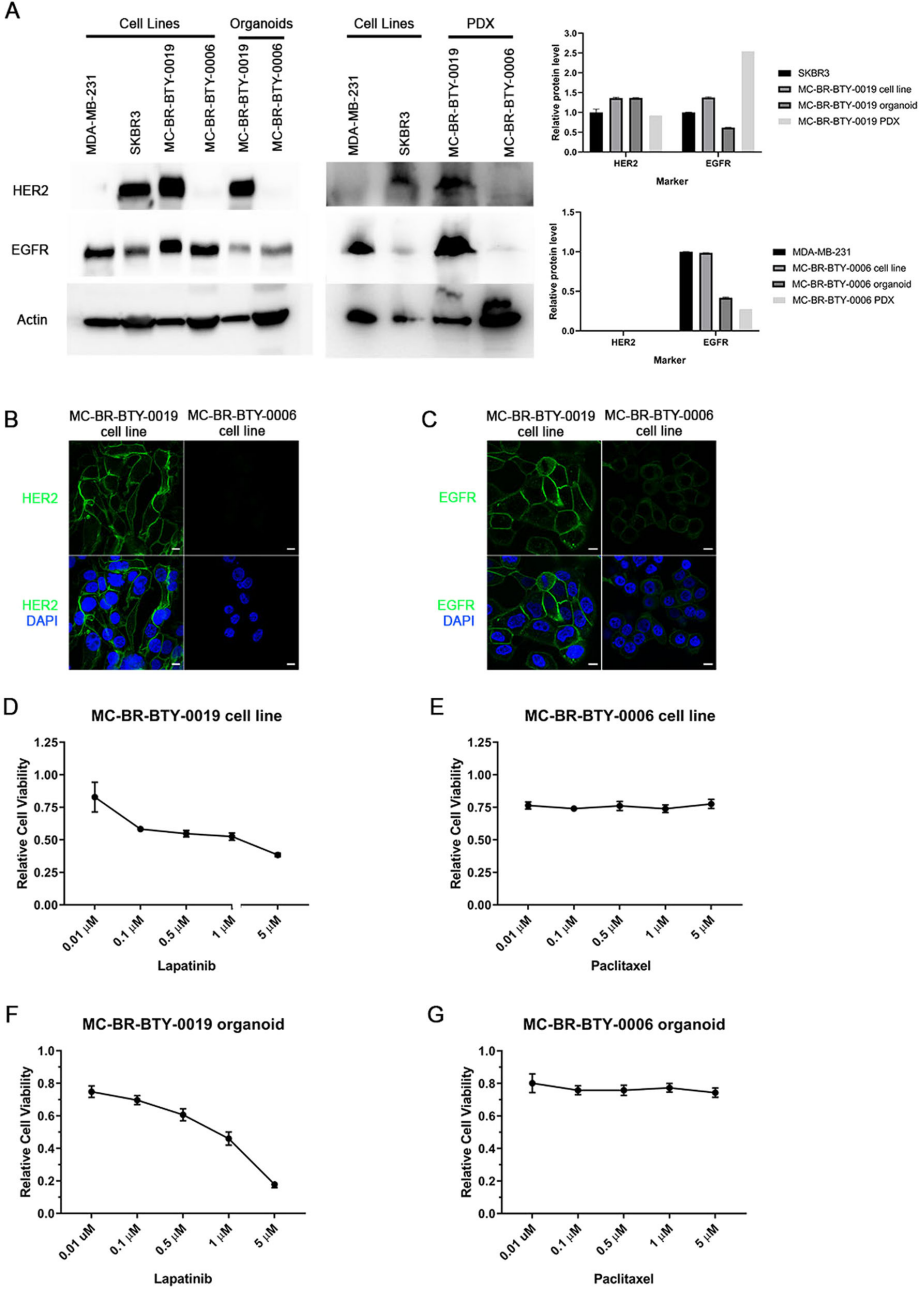
Cell lines, MC-BR-BTY-0019 and MC-BR-BTY-0006 maintain the subtype characterization and drug response consistent with PDX-derived organoid and PDX tumor. **A** Western blot and quantification of the levels of HER2, EGFR, and beta-actin in breast cancer cell lines, newly established cell lines, their corresponding PDX-derived organoids and PDX tumors. Expression of HER2 and EGFR for MC-BR-BTY-0019 cell line, organoid, and PDX was normalized to beta-actin and then SKBR3. Expression of HER2 and EGFR for MC-BR-BTY-0006 cell line, organoid, and PDX was normalized to beta-actin and then MDA-MB-231. All blots are derived from the same experiment and were processed in parallel. **B** MC-BR-BTY-0019 and MC-BR-BTY-0006 cell lines were stained with HER2 and DAPI and images were taken at ×100. Scale bar represents 10 μm. **C** MC-BR-BTY-0019 and MC-BR-BTY-0006 cell lines were stained with EGFR and DAPI and images were taken at ×100. Scale bar represents 10 μm. **D** 10,000 cells/well of MC-BR-BTY-0019 cell line were seeded in 96-well plates in triplicates, treated with indicated concentrations of lapatinib for 48 h, and cell viability was measured by MTS assay and normalized to DMSO. IC_50_ = 438.531 nM. **E** 10,000 cells/well of MC-BR-BTY-0006 cell line were seeded in 96-well plates in triplicates, treated with paclitaxel at indicated concentrations for 48 h, and cell viability was measured by MTS assay and normalized to DMSO. IC_50_ = Indeterminate. **F**, **G** PDX tumors were collected when the tumor achieved 10–20 mm in diameter, and primary breast cancer cells were isolated after dissociation of tumor tissue from mouse cells, as previously described^7^. MC-BR-BTY-0019 PDX-derived organoids were generated by seeding 10,000 cells of the primary breast cancer single-cell suspension per well in Nanoculture plates in triplicates and treated with indicated concentrations of lapatinib for 5 days (IC_50_ = 979.49 nM). MC-BR-BTY-0006 PDX-derived organoids were treated with indicated concentrations of paclitaxel for 5 days (IC_50_ = Indeterminate). Organoid viability was measured using 3D Cell TiterGlo viability assay, normalized to DMSO, and plotted using GraphPad PRISM software. Error bars represent SEM.

Breast cancer cell lines are commonly categorized by the expression of clinical corresponding molecular markers, such as estrogen receptor (ER), ErbB2 receptor tyrosine kinase 2 (HER2), and epidermal growth factor receptor (EGFR)^16^. The two established cell lines MC-BR-BTY-0019 and MC-BR-BTY-0006 were originally derived from HER2+ and triple-negative breast cancer, respectively. To characterize the molecular subtype of the established cell lines, western blot was performed to examine the level of HER2 and EGFR. We compared the protein levels between the two newly established cell lines with their corresponding PDX-derived organoid, PDX tumor tissues and two commonly studied breast cancer cell lines (Fig. 3A).

The cell line MC-BR-BTY-0019, and its PDX-derived organoid expressed HER2 at similar levels in comparison with well studied HER2+ cell line, SKBR3, while slightly decreased in the PDX tumor. MC-BR-BTY-0006 was HER2-negative across all three culture types (cell line, organoid, PDX). The relative abundance of EGFR in MC-BR-BTY-0019 and MC-BR-BTY-0006 cell lines was comparable to SKBR3 and MDA-MB-231, respectively, but significantly higher than the corresponding PDX-derived organoid. For MC-BR-BTY-0006, EGFR levels were lowest in its derived PDX tumor tissue, but highest for MC-BR-BTY-0019. Immunofluorescent staining in Fig. 3B, C demonstrated HER2 and EGFR were specifically enriched in the plasma membrane in the two established cell lines. These demonstrated that MC-BR-BTY-0019 and MC-BR-BTY-0006 retained the subtype feature (HER2 status) as well as EGFR status, even though the expression of both HER2 and EGFR are slightly different between cell lines, organoids and PDX tumors.

Lapatinib is a small molecule kinase inhibitor of HER2 and EGFR that has been clinically used to treat HER2+ breast cancer^17^. Paclitaxel is a standard chemotherapy drug targeting microtubule formation thus inhibiting cancer cell mitosis, and has been clinically used to treat all breast cancer subtypes, including triple-negative breast cancer^18^. In order to examine whether the response to treatment was similar between cell line and PDX-derived organoid in vitro, the cell lines, as well as the organoids, were treated with specific targeted drugs, lapatinib in HER2+ MC-BR-BTY-0019 and paclitaxel in triple-negative MC-BR-BTY-0006. MC-BR-BTY-0019 cell line was sensitive to lapatinib treatment, consistent with PDX-derived organoid response with an IC50 of 438.531 and 979.49 nM, respectively, for cell line and organoids (Fig. 3D, F). MC-BR-BTY-0006 cell line did not exhibit sensitivity to paclitaxel treatment up to maximum concentration (5 μM), also consistent with its corresponding PDX-derived organoids (Fig. 3E, G).

### The two established breast cancer cell lines maintained in vivo tumorigenesis capability, similar histologic features, and similar in vivo drug response as compared to parental PDX models

**Fig. 4:**
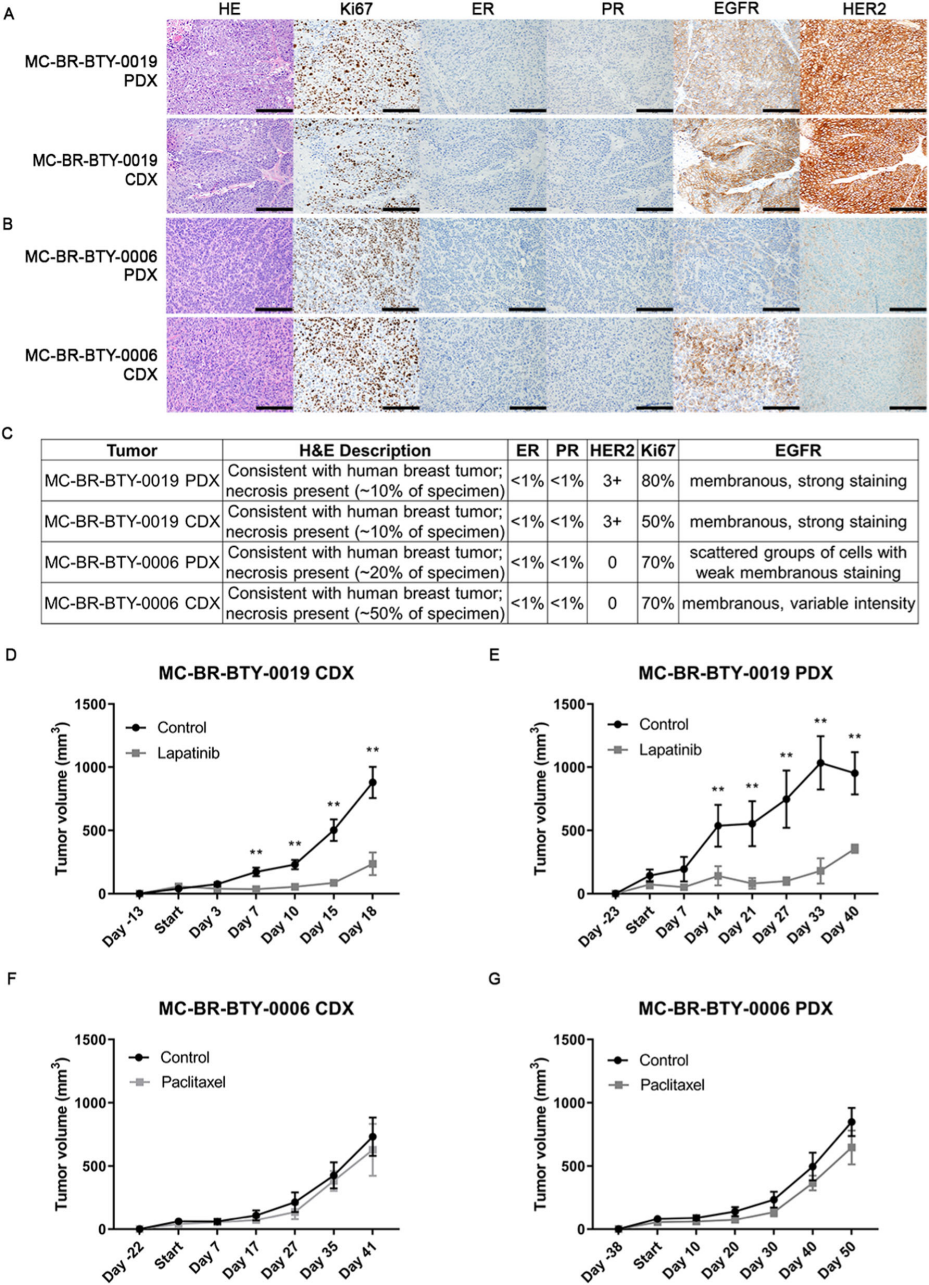
The two established breast cancer cell lines form CDX tumors in vivo, maintain similar pathology features as their corresponding PDX tumors and show similar drug response in vivo. **A**, **B** Pathology staining of HE, Ki67, ER, PR, EGFR, and HER2 was performed in both PDX tumor tissue and CDX tumor formed from MC-BR-BTY-0019 cell line (**A**) and MC-BR-BTY-0006 (**B**). Images taken at ×200; Scale bar represents 200 μm. **C** Detailed description of the pathology staining features of tumor tissue of the PDX and CDX is summarized for MC-BR-BTY-0019 and MC-BR-BTY-0006. **D** MC-BR-BTY-0019 CDX in vivo tumors were formed by injecting 2 million cells of MC-BR-BTY-0019 cell line into 10 NSG mice, which were randomized when tumor achieved 50–100 mm^3^ in volume, and treated with either control (*n* = 5, vehicle, 5 days/week) or lapatinib (*n* = 5, 50 mg/kg, 5 days/week) for 3 weeks. Tumor volume was measured at indicated time points and plotted using GraphPad PRISM software. Error bars represent SEM. **p < 0.01, 2 tailed Student’s *t*-test comparing vehicle to drug treatment. **E** MC-BR-BTY-0019 PDX tumor was formed by injecting earlier passage of PDX tumor into flank of 15 NSG mice, 13 mice with similar size of tumors were randomized when tumor achieved 50–100 mm^3^ in volume and treated with either control (*n* = 6, vehicle, 5 days/week) or lapatinib (*n* = 7, 50 mg/kg, 5 days/week) for 3 weeks. Tumor volume was measured at indicated time points and plotted using GraphPad PRISM software. Error bars represent SEM. **p < 0.01, 2 tailed Student’s *t*-test comparing vehicle to drug treatment. **F** MC-BR-BTY-0006 CDX in vivo tumors were formed by injecting 5 million cells of MC-BR-BTY-0006 cell line into 10 NSG mice, which were randomized when the tumor reached 50–100 mm^3^ in volume, and treated with either control (*n* = 5, vehicle, 2 times/week) or paclitaxel (*n* = 5, 12.5 mg/kg, 2 times/week) for 3 weeks. Tumor volume was measured at indicated time points and plotted using GraphPad PRISM software. Error bars represent SEM. *p*-value = ns, 2 tailed Student’s *t*-test comparing vehicle to drug treatment. **G** MC-BR-BTY-0006 PDX tumor was formed by injecting earlier passage of PDX tumor into flank of 15 NSG mice, 12 mice with similar size of tumor were randomized when tumor reached 50–100 mm^3^ in volume, and treated with either control (*n* = 6, vehicle, 2 times/week) or paclitaxel (*n* = 6, 12.5 mg/kg, 2 times/week) for 3 weeks. Tumor volume was measured at indicated time points and plotted using GraphPad PRISM software. Error bars represent SEM. *p*-value = ns, 2 tailed Student’s *t*-test comparing vehicle to drug treatment.

Breast cancer PDX are valuable tools to study drug response in vivo, however, the turnaround time to get the tumor to grow in mice even with an established tumor line is variable; usually 1–5 months depending on the characteristics of the tumor^5^. On the other hand, immortalized cell lines have long been used to develop cell line-derived xenografts (CDX), forming tumors within weeks^19^. However, even with commonly used breast cancer cell lines, not all of them can be engrafted and grown into tumor^20^. When our two newly developed cell lines, MC-BR-BTY-0019 and MC-BR-BTY-0006 were injected into the right flank of the NSG mice, tumors formed after about 2 and 3 weeks, respectively (Fig. 4D, F). We further characterized and compared the pathological features of the CDX versus PDX tumor using H&E and immunohistochemistry staining against Ki67, ER, PR, HER2, and EGFR (Fig. 4A–C). Both CDX tumors maintained similar histologic features as their corresponding PDX tumors.

We also compared the growth rate and drug response of the CDX and PDX tumors in vivo. The MC-BR-BTY-0019 and MC-BR-BTY-0006 PDX models achieved a tumor volume of 50–100 mm^3^ in 3 and 5 weeks, respectively (Fig. 4E, G), while MC-BR-BTY-0019 and MC-BR-BTY-0006 CDX models achieved a similar tumor volume in 2 and 3 weeks, respectively. Next, the MC-BR-BTY-0019 CDX and PDX models were treated with lapatinib (50 mg/kg, 5 days/week, i.p.) for 3 weeks, and the MC-BR-BTY-0006 CDX and PDX models were treated with paclitaxel (12.5 mg/kg, 2 times/week, i.p.) for 3 weeks. Consistent with the cell line and organoid-based drug response (Fig. 3D–G), both the MC-BR-BTY-0019 CDX and PDX tumors were sensitive to lapatinib treatment, while the MC-BR-BTY-0006 CDX and PDX tumors were resistant to paclitaxel treatment (Fig. 4D–G). Here we demonstrate that the established cell lines not only induced tumor formation in vivo in a shorter time period compared to their corresponding PDX tumors but also maintained the same drug response to major commonly used drugs for these two subtypes. These two cell lines could thus provide additional cell models for mechanistic studies involved in tumorigenesis as well as drug response while still retaining characteristics of the PDX tumors.

### Molecular profiles of cell lines and corresponding PDX tumors

**Fig. 5:**
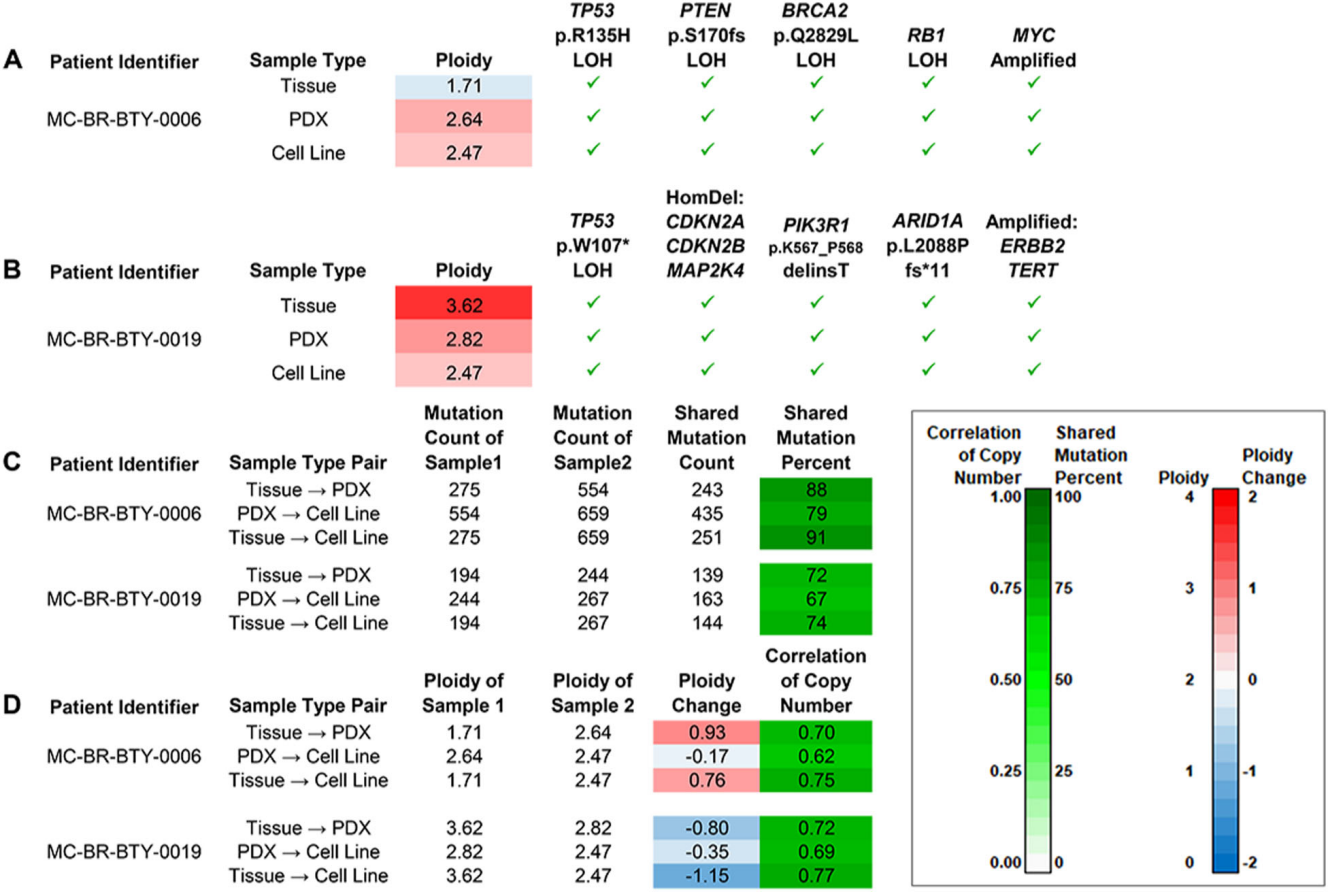
Exome sequencing reveals several patient-specific cancer driver mutations and an evolutionary trajectory that features their preservation in the presence of somatic changes suggestive of genome instability. **A** Oncogenic mutations in five cancer genes were detected in all three MC-BR-BTY-0006 tumor samples. Pathogenic loss-of-function somatic SNVs were detected in one allele of each of the genes TP53, PTEN, and BRCA2 as well as the other allele (loss of heterozygosity, or LOH). Thus, loss of function of both copies of tumor-suppressor genes TP53, PTEN, and BRCA2 could be inferred. RB1 loss of heterozygosity (LOH) and amplification of MYC were other oncogenic events observed. **B** Oncogenic mutations in eight cancer genes were detected in all three MC-BR-BTY-0019 tumor samples. A different pathogenic loss-of-function mutation in TP53 for cell line, but was again observed alongside LOH. So, loss of function of both copies can be inferred for TP53 as well as for three other tumor suppressor genes, CDKN2A, the adjacent CDKN2B, and MAP2K4, due to homozygous deletion (HomDel). Mutations were also observed in PIK3R1 and ARID1A. The mutations affect sites in regions frequently altered in cancer. Amplifications of TERT and ERBB2, or HER2, were also detected, with particularly high amplification for ERBB2. **C** Of the somatic SNV/INDELs detected in the patient tumor tissue samples, proportions detected in PDXs and cell lines were high (≥72%) for both patients. Of those mutations detected in PDXs, proportions detected in cell lines were also high (79%, 67%). **D** Changes in ploidy by sample type pair. For each patient, the absolute differences were nearly 1 copy from tissue to PDX with a Tissue→PDX increase observed for MC-BR-BTY-0006 and a Tissue→PDX decrease observed for MC-BR-BTY-0019. Despite these changes, the profiles of within-sample relative copy numbers for Tissue and PDX still showed similarity as the correlation of copy number was still 0.70 or greater. Slight decreases in ploidy were observed from PDX to cell line for both patients.

We have shown that cell lines derived from PDX tumors have comparable pathology as well as drug response phenotypes when compared with their corresponding PDX in vitro and in vivo. We took a step further to examine the tumor genetic alterations in the cell lines, PDX models, and original patient tumors. Exome sequencing was performed for both cell lines and their corresponding patient tumor tissue and PDX samples. After comparing between samples, several SNV/INDEL mutations and copy number alterations in important tumor suppressor genes, genes regulating protein stability, and oncogenes were observed uniformly across the different sample types. Figure 5 lists those genes that were significantly affected in each patient model. At least five such genes were impacted for MC-BR-BTY-0006 (Fig. 5A), and at least eight genes were affected for MC-BR-BTY-0019 (Fig. 5B). For SNV/INDELs, while a substantial number of new mutations were detected from tissue to PDX and from PDX to cell line, a large proportion of the somatic variants of the original sample were retained (67–91%) (Fig. 5C). For both MC-BR-BTY-0006 and MC-BR-BTY-0019, we observed that copy number changes resulted in changes in ploidy of nearly 1 in absolute value from tissue to PDX. An increase was observed for MC-BR-BTY-0006 (Fig. 5A, D, Supplemental Fig. 3A), and a decrease was observed for MC-BR-BTY-0019 (Fig. 5B, D, Supplemental Fig. 3B). These changes are suggestive of genomic instability, to which the observed alterations to TP53 (MC-BR-BTY-0006 (Fig. 5A) and MC-BR-BTY-0019 (Fig. 5B)), BRCA2 (MC-BR-BTY-0006 (Fig. 5A)), and ARID1A (MC-BR-BTY-0019 (Fig. 5B)) are likely contributors^21,22^. We also observed ERBB2 amplification in the MC-BR-BTY-0019 samples (Fig. 5B). An association between ERBB2 amplification and chromothripsis has been identified in breast cancer^23^. Chromothripsis, if present in the MC-BR-BTY-0019 tumor, would also be expected to contribute to genomic instability. Copy number profiles still exhibited substantial similarity across sample types with intra-patient correlations ranging from 0.62 to 0.77 (Fig. 5D). In summary, for each patient, there is uniform observation across sample types of a number of important tumorigenic alterations. At the same time, we also find evidence that the tumors continue to evolve between models, which is likely due, at least in part, to alterations known to be associated with genomic instability.

## Discussion

PDX have emerged as a widely used resource to recapitulate patient tumor behavior, and have become a valuable tool for cancer research. However, PDX mouse models are particularly difficult for functional or mechanistic studies due to logistical and financial reasons— limiting their application. Deriving immortalized breast cancer cell lines from PDX tumors could provide researchers with an additional laboratory resource to efficiently perform high throughput studies, such as drug screening, without the burdens associated with PDX mouse models.

In our current study, breast cancer PDX models including two ER+, four triple-negative, and three HER2+ subtypes were used in an attempt to establish immortalized primary breast cancer cell lines. In general, PDX models that grew relatively fast in vivo and contained less stroma were easier to be established into breast cancer cell lines. However, PDX models that failed to transform into immortalized cell lines exhibited different characteristics in cell culture. Two ER+ cell lines initially proliferated in cell culture after being isolated from ER+ PDX tumors, however, they failed to proliferate once they were dissociated from the flasks. To overcome this, we attempted to optimize various culture conditions including modulating oxygen or estrogen levels, using different cell dissociation agents, or supplementing media with additional growth factors; however, none of these attempts improved our success rate. Most of the cells we tried to develop were viable for several weeks but failed to sufficiently proliferate to produce enough materials for characterization and downstream studies. Aside from using PDX-established tumors, we also attempted to establish breast cancer cell lines directly from patient biopsy samples. However, due to the limited amount of tissue available, a limited success rate was observed in our attempt to develop cell lines using primary biopsy samples, similar to what was described by others^11^. In our study, we achieved a 22% success rate of cell line immortalization from PDX tumors. Nevertheless, in future, it would be worthy to explore the feasibility of establishing cancer cell lines from bulk surgical samples where a larger amount of tumor cells could be obtained.

Phenotypic profiling of our two established cell lines revealed several interesting features compared to well-studied cell lines. We observed that even after establishing the immortalized cell lines, cells with lower confluency proliferated at a relatively slower rate, emphasizing the role of cell–cell interaction in maintaining faster cell proliferation. The doubling time for MC-BR-BTY-0019 was about 24–48 h, shorter than the commonly used HER2+ breast cancer cell lines such as SKBR3 (48–72 h (DSMZ)), MDA-MB-453 (50–60 h (DSMZ)), BT474 (100 h (DSMZ)), and MDA-MB-361 (103.2 h)^24^. The doubling time for MC-BR-BTY-0006 was about 48–72 h, comparable or slightly longer than typical triple-negative breast cancer cell lines MDA-MB-231 (38 h (ATCC)), BT20 (70 h)^25^, BT549 (53.9 h (NCI-DTP)), and Hs578T (53.8 h (NCI-DTP)). STR profiling for the two cell lines demonstrated they were unique cell lines without any similarity to existing breast cancer cell lines (Fig. 1D, E), and matched their PDX of origin (Fig. 1F, G). While it would have been more convincing to perform comparisons with the original patient tumor, a limited quantity of patient biopsy samples prevented us from verifying a tumor profile match in this study.

The further characterization of these two cell lines revealed significantly abnormal karyotypes. The data we have obtained from the karyotypes indicated these two cell lines had clonal variation within the established cell lines. For MC-BR-BTY-0019, 11 among the 20 examined metaphases represented a tetraploid subclone and MC-BR-BTY-0006 presented clonal structural rearrangements (Fig. 2). We cannot rule out the possibility that there might be more clonal heterogeneity that could not be detected by the karyotype since only 20 cells were examined in our study. In the future, single-cell RNA sequencing of the established cell lines could provide more reliable answers to the level of heterogeneity of the cells.

The molecular subtype of these two cell lines was compared by detecting HER2, EGFR proteins in PDX-derived organoids and PDX tumor tissues. The results demonstrated that the HER2 expression was comparable between cell line, organoid, and PDX tumor tissue for MC-BR-BTY-0019 and MC-BR-BTY-0006 as shown in Fig. 3. Similar levels of HER2 positivity were also observed between CDX and PDX tumors (Fig. 4). However, we observed variation in EGFR expression levels between different models. EGFR for MC-BR-BTY-0006 and MC-BR-BTY-0019 were two-fold higher in the cell lines than their respective PDX-derived organoid (Fig. 3A). Similar differences in EGFR levels have been previously observed when comparing 2D and 3D lung adenocarcinomas^26^. Therefore, we hypothesize our observations may be due to a combination of differing dimensional architecture between culture methods and selective pressures placed on the cell population by the culture media to select high EGFR-expressing cells—a phenomenon that has been observed by others, suggesting the cell culture environment plays an important role in cell selection process^27–29^. For MC-BR-BTY-0019, comparisons of our IHC data (Fig. 4A) and western blots (Fig. 3A) between cell line, CDX and PDX suggest EGFR levels were higher in the cell lines and PDX compared to the organoid. Despite this variation, the response to Lapatinib (a dual inhibitor of EGFR and HER2) was consistent among all three models (Figs. 3D, F and 4D, E). This finding also supports a previous finding that HER2-positive breast cancer response to Lapatinib is EGFR-independent^30^.

Cell lines that can form tumors in vivo play an important role in translational studies. The success of many breast cancer cell lines to form CDX tumors is in part due to their origin from metastatic lesions^31^. Here we demonstrated the success in forming CDX tumors using two cell lines established from primary breast cancer tumors. Currently, MCF-7 and T47D are two widely used ER+ luminal A cell lines that can form tumors in vivo in the presence of estrogen^20^. Cell lines representing the HER2+ subtype, such as SKBR3 and MDA-MB-453, have poor tumorigenic potential^20^. Here we have established MC-BR-BTY-0019, which is one HER2+ breast cancer cell line that can form CDX tumors in NSG mice in 2 weeks (Fig. 4D). As for triple-negative breast cancer in vivo models, MDA-MB-468 and MDA-MB-231 are the most commonly used cell lines^20^. Our established MC-BR-BTY-0006 cell line can form CDX tumors after 3 weeks, further increasing the pool of available triple-negative cell lines that can form CDX tumors in vivo. The CDX took a shorter time period to form a measurable tumor after implantation compared to PDX and had a much faster tumor growth rate once it reached a volume of 500 mm^3^. Since this study primarily focused on demonstrating the feasibility of establishing PDX-derived breast cancer cell lines and the basic characteristics of these cell lines, we only tested the most commonly used drugs in HER2+ and triple-negative breast cancer cells—lapatinib and paclitaxel, respectively. Despite some observed differences in pathology between the CDX and PDX tumors, their response to drug treatment remained consistent (Fig. 4). In future studies, it would be interesting to test a wider panel of drugs for these two cell lines to further extend the applications of these two cell lines in drug screening and understanding drug mechanisms of action.

The exome sequencing results further confirmed that the cell lines largely retained the same genomic profile compared to the PDX tumors and the patient tumors (Fig. 5). We have shown that the cell lines derived from PDX tumors have retained a majority of the somatic SNV/INDEL mutations that we detected in the patient tumor tissue samples (Fig. 5C). However, we also observed additional somatic SNV/INDEL mutations (Fig. 5C). There are different possible explanations for this. The high tumor purity of the cell line sample makes it possible to detect subclonal somatic mutations for which there may have been no alternate-allele supporting reads in the patient tumor tissue sample. The additional large-scale events arising in the PDX sample and maintained in the cell line could also have enabled the detection of additional somatic mutations with low alternate-allele frequency in patients’ genomic regions. Certainly, there are also novel somatic mutations that might arise due to tumor evolution. We cannot dismiss the possibility that some detected mutations are artifacts that could have arisen in sample processing or analysis. Further research will allow for better characterization of these additional somatic SNV/INDEL mutations and assessment of their impact on the tumor.

We have also observed that the cell lines derived from PDX tumors experience ploidy change from patient tumor tissue to PDX tumors (Fig. 5A), which was largely maintained in the PDX-derived cell lines. Such changes are not unexpected in the evolution of a tumor^32^. From our analysis, we inferred that the copy number alteration profiles are largely defined by a combination of the patient tumor tissue profile and a single copy increase. Further research is required to better understand the implication of this phenomenon on tumor growth and interpretation of results obtained between cell lines, PDX tumors and patients’ data. Therefore, cautions should also be taken as our data suggested that there were certain differences between CDX and PDX. The advantage and disadvantage of using CDX and PDX should be considered in choosing models and data interpretation in translational drug developments. Finally, the availability of those cell lines will also have greater value for in vitro mechanistic applications.

In conclusion, primary breast cancer cell lines can be derived from PDX maintained in NSG mice. The established cell lines will provide convenient tools for further drug screening and mechanistic study in vitro and in vivo*.* However, like any in vitro models, caution should be taken to interpret the results obtained from the cell lines and their applications to patients.

## Methods

### Establishment of primary breast cancer cell line

Breast tumor tissue obtained from a percutaneous biopsy of patients with primary non-metastatic breast cancer recruited on a prospective trial, BEAUTY^5,6^ was implanted into the mammary fat pad of 6–8-week-old female NSG NOD.Cg-*Prkdc*^*scid*^ *Il2rg*^*tm1Wjl*^/SzJ (Stock No. 005557) female mice and maintained as PDX as described in a previous study^5,6^. No human subject was involved in this particular study. All protocols for animal studies have been approved by the Institutional Animal Care and Use Committee (IACUC) of Mayo Clinic. PDX tumors were collected when the tumor grew to ~10–20 mm diameter. Primary breast cancer cells were isolated after dissociation of tumor tissue from mouse cells, as previously described^7^. A single-cell suspension of primary breast cancer cells was initially cultured in mouse embryonic fibroblast (MEF) media (DMEM with 10% fetal bovine serum (FBS) supplemented with glutamax, MEM NEAA, sodium pyruvate, and 5 μM Y-27632-inhibitor (Tocris)). Once the primary cancer cells were established after two passages, cells were maintained in DMEM with 10% FBS supplemented with Anti–Anti (Gibco).

### Immunofluorescence

MC-BR-BTY-0019 and MC-BR-BTY-0006 cell lines were plated onto 16-well CultureWell™ chambered coverglass (Molecular Probe C37000) with 20,000 cells per well. Cells were fixed with 4% formaldehyde the next day and treated with or without 0.1% Triton X-100. The cells were then blocked with 3% BSA for 30 min and incubated with either HER2 or EGFR antibodies overnight at 4 °C. Cells were incubated with appropriate Alexa Fluor antibody for one hour at room temperature and stained with DAPI before visualization by Zeiss LSM 780 confocal microscope at ×100 magnification. Antibodies against HER2 (#2165; 1:50) and EGFR (#4267; 1:50) were purchased from Cell Signaling. Secondary Antibody, Alexa Fluor 488 (R37116; 1:1000) was purchased from Invitrogen.

### Western blot

MC-BR-BTY-0019 and MC-BR-BTY-0006 cell lines were lysed by Laemmli sample buffer. 300 mg of PDX tumor was placed into microcentrifuge tubes containing 300 μL NETN lysis buffer (100 mM NaCl, 20 mM Tris–HCl pH = 0.8, 0.5 mM EDTA, NP-40) containing phosphatase and proteasome cocktail inhibitors and 0.5 mm RNase-free zirconium oxide beads (Next Advance). Tumors were lysed by placing tubes into a bullet blender for 10 minutes at 4 °C. The concentration of cell lysates was then quantified via Bradford assay and mixed with Laemmli sample buffer containing 10% 2-ME and boiled for 10 min. Equal amounts of protein of each cell line were separated on 4–20% SDS-polyacrylamide gels. Proteins were transferred to PVDF membranes using a semi-dry Bio-Rad Trans-blot apparatus with Trans-Blot^®^ Turbo™ RTA Mini PVDF Transfer Kit (BioRad #1704272). The membrane was then incubated with the appropriate antibody in TBS-T (1× TBS with 0.1% Tween-20) containing 5% non-fat dry milk overnight at 4 °C followed by incubating with the horseradish peroxidase (HRP)-conjugated secondary antibody for one hour. Proteins were detected using the SuperSignal™ West Dura Extended Duration Substrate (BioRad Cat#34075). Primary antibodies against beta-Actin (#4970; 1:1000), HER2/ErbB2 (#2248; 1:200) and EGFR (#4267; 1:1000) were purchased from Cell Signaling. Secondary horseradish peroxidase-linked anti-mouse (#7076s; 1:5000) and anti-rabbit (#7074P2; 1:5000) IgG antibodies were purchased from Cell Signaling.

### Drug response of cell line and PDX-derived organoid

MC-BR-BTY-0019 and MC-BR-BTY-0006 cell lines were seeded in DMEM-10% FBS in a 96-well cell culture plate with 10,000 cells per well. After cell attachment, various concentrations of lapatinib (Selleckchem) and paclitaxel (Selleckchem) were used to treat the cells. Following 48-h treatments, MTS (Sigma) was added to the wells according to the manufacturer’s instructions to obtain dose–response curve. MC-BR-BTY-0019 and MC-BR-BTY-0006 organoids were grown from corresponding PDX tumors freshly harvested from mice and cultured in MEF media with 10,000 cells per well in nanoculture plates. Briefly, PDX tumors were collected when the tumor grew to ~10–20 mm diameter. Primary breast cancer cells were isolated after dissociation of tumor tissue from mouse cells, as previously described^7^. A single-cell suspension of primary breast cancer cells was initially cultured in MEF media (DMEM with 10% FBS supplemented with glutamax, MEM NEAA, sodium pyruvate, and 5 μM Y-27632 ROCK inhibitor (Tocris)). ROCK inhibitor was removed from media by changing media one week before drug treatment. Cells were treated with various concentrations of lapatinib or paclitaxel for 5 days. 3D cell TiterGlo kit (Promega) was used to measure 3D culture viability according to the manufacturer’s instructions to obtain dose–response curves.

### In vivo drug response in PDX and cell line re-grafted mice

Two million cells of MC-BR-BTY-0019 or five million cells of MC-BR-BTY-0006 were injected into the right flank region of 20, 6–8-week-old female NSG mice, and tumors were monitored weekly for the first two weeks and every other day thereafter for tumor growth. MC-BR-BTY-0019 developed detectable tumors in 2 weeks, and MC-BR-BTY-0006 developed detectable tumors in 3–4 weeks. When the tumor volume reached ~50–100 mm^3^, mice bearing MC-BR-BTY-0019 were randomized into two groups and treated with either vehicle control or lapatinib at a dose of 50 mg/kg (i.p. 5 days/week) for 3 weeks. Mice bearing MC-BR-BTY-0006 were randomized into two groups and treated with paclitaxel at a dose of 12.5 mg/kg (i.p. 2 times/week) for 3 weeks. The two PDX tumors were implanted into 30, 6–8-week-old female NSG mice, respectively, as previously described^5^ followed by the same treatments as the cell line re-grafted mice. Mice in which did not take tumor were excluded. Study was not blinded. Tumor size was monitored twice every week and mice were euthanized when they met the euthanization criterion. Error bars represent SEM.

### Cell morphology and cell doubling time

Images of established cell lines (MC-BR-BTY-0019 and MC-BR-BTY-0006) were taken using Invitrogen microscopy at ×20 at passage 5. The doubling time of MC-BR-BTY-0019 and MC-BR-BTY-0006 cell lines at passage 5 was determined by counting cells every 24 h for 5 days after plating 30,000 cells and 85,000 cells in 24-well plates for MC-BR-BTY-0019 and MC-BR-BTY-0006, respectively. Cells were trypsinized and counted using trypan blue staining the live cells every 24 h. Growth curves of each cell line were plotted after normalizing to day 1, and the doubling time was calculated using exponential regression of the cell growth curve with the software, Prism Graphpad. Error bars represent SEM.

### Karyotyping

Karyotyping of two cells of each cell line, MC-BR-BTY-0019 and MC-BR-BTY-0006, were performed after G-band staining, and analysis was done by a certified cytogenetist at Mayo Clinic. Briefly, 1 million cells were cultured in a T75 flask, and Colcemid (1 µg/mL) was added to the medium for 14–16 h. Cells were detached with TrypLE Express for 5–10 min and pelleted down after centrifugation. 5 mL of 50/50 mixture of 0.8 M sodium citrate hypotonic solution and 0.075 M potassium chloride hypotonic solution was added to the cell pellets, followed by adding 2 mL of 3:1 methanol to glacial acetic acid fixative. Cells were pelleted down and fixed with 10 mL of 3:1 methanol to glacial acetic acid. Slides were incubated in a Thermotron slide drying chamber at 25 °C and 65% relative humidity. Cells were then processed for G-band staining. 20 cells were analyzed with 2 cells fully Karyotyped.

### STR profiling

STR profiling was performed by LabCorp Genetica using genomic DNA obtained from MC-BR-BTY-0019 and MC-BR-BTY-0006 cell lines and PDX. 15 autosomal STR loci and the gender identity locus amelogenin were utilized to determine the uniqueness of cell lines when compared to ATCC, DSMZ, JCRB databases. Mouse loci were utilized to detect mouse cell contamination.

### Pathology of PDX and CDX tumors

Early passage (within passage 10) PDX tumor samples were collected after euthanizing the tumor-bearing mice. Half of the tumor was fixed in formalin for pathology. The other half was used for tumor cell line establishment. Morphologic review of hematoxylin and eosin-stained sections from each PDX tumor was performed along with a review of immunohistochemically stained slides to ensure that the PDX tumor retained features of the original patient tumor. After cell lines were established, they were reseeded in 150 mm dishes for 3 days. Cells were trypsinized after washing with 1× PBS, counted and resuspended into DMEM with 10% FBS. CDX tumors were collected and fixed in formalin for pathology. Sections from each tumor were stained in the Pathology Research core using antibodies against ER (1D5 clone, Dako), PR (PgR 363 clone, Dako), Ki67 (MIB-1 clone, Dako), and EGFR (D38B1, Cell Signaling Technology), and in the clinical IHC laboratory at Mayo Clinic against HER2 (Ventana Pathway using the 4B5 clone, Ventana Medical Systems). ER and PR were scored as follows: negative (<1% reactive cells), focally positive (1–10% reactive cells), and positive (>10% reactive cells). HER2 was scored following CAP/ASCO guidelines for clinical testing. Ki67 was scored approximating the percentage of positive nuclei to the nearest 10%. EGFR was scored as negative, weak, moderate, or strong membranous staining. The pathology of parental PDX tumors and CDX tumors were compared to demonstrate their similarities and differences.

### Isolation of genomic DNA from cell lines and PDX

Genomic DNA was isolated from cultured cell lines MC-BR-BTY-0019 and MC-BR-BTY-0006 using the QIAGEN Blood and Cell Culture DNA kits. Briefly, 5 × 10^6^ cells were washed in 1× PBS and resuspended into 500 μL 1× PBS. Subsequently, 500 μL of ice-cold better C1 and 1.5 mL of ice-cold distilled water were added, and the lysed cells were centrifuged at 4 °C for 15 min at 1300×*g*. The supernatant was discarded and the pellet was resuspended in 250 μL of ice-cold buffer C1 and 750 μL of ice-cold distilled water and mixed by vortexing. The nuclear pellet was obtained after centrifuging the mixture again at 4 °C for 15 min at 1300×*g* and resuspended into 1 ml of Buffer G2 by vortexing for 10–30 s at maximum speed to lyse the nuclei and denature proteins. Finally, 25 μL of Qiagen protease cocktail was added and the mixture was incubated at 50 °C for 60 min to further digest the denatured proteins. All subsequent steps were performed according to the manufacturer’s Genomic-tip protocol. For PDX tissues, genomic DNA was isolated after homogenization using QIAGEN DNeasy Blood & Tissue Kit following the manufacturer’s protocol.

### Whole-exome sequencing

DNA-sequencing was performed by Mayo Clinic Core facility using Sure Select XT Whole Exon Capture v5 + UTRs 75 MB and Illumina HiSeq 4000 Paired-End Sequencing. Paired-end libraries were prepared using 1.0 μg of genomic DNA following the manufacturer’s protocol (Agilent) using the Agilent Bravo liquid handler. Whole exon capture was carried out using 750 ng of the prepped library following the protocol for Agilent’s SureSelect Human All Exon v5 + UTRs 75 MB kit. The purified capture products are then amplified using the SureSelect Post-Capture Indexing forward and Index PCR reverse primers (Agilent) for 12 cycles. The concentration and size distribution of the completed libraries was determined using an Agilent Bioanalyzer DNA 1000 chip (Santa Clara, CA) and Qubit fluorometry (Invitrogen, Carlsbad, CA). Libraries were sequenced at an average coverage of ~250× following Illumina’s standard protocol using the Illumina cBot and HiSeq 3000/4000 PE Cluster Kit. The flow cells were sequenced as 150 × 2 paired-end reads on an Illumina HiSeq 4000 using HiSeq 3000/4000 sequencing kits and HCS v3.3.52 collection software. Base-calling is performed using Illumina’s RTA version 2.7.3.

### Exome-sequencing: alignment, pre-processing, and exome coverage

We sequenced DNA extracted from samples of blood (used as the normal sample), tumor tissue, PDX, and PDX-derived immortalized cell lines for two patients (MC-BR-BTY-0019 and MC-BR-BTY-0006) of the BEAUTY study^6^. All of the sequence results used in the subsequent analysis passed quality controls according to the Illumina manufacturer (HiSeq2000 for the normal and tissue; HiSeq4000 for the PDX and cell line) and also passed the quality control measures of the FASTQC software. To map reads to the human genome, we used the BWA-MEM (v 0.7.10) alignment program within the Mayo Clinic’s DNA sequencing analysis pipeline, GenomeGPS (v4.0.1)^33^. We were able to map reads to the human reference genome (UCSC hg38 with alternate contigs removed) at consistently high rates, ranging between 98.7% and 99.9% (Supplemental Data 1). Realignment and recalibration were performed using GATK (version 3.4–46)^34^. Variant quality score recalibration was done using GATK. Duplicate reads were identified and marked using the Picard MarkDuplicates program. Agilent SureSelect Human All Exon + UTRs capture kits were used for all samples (v4 for normal and tissue; v5 for PDX and cell line). For each sample, the percentages of the intersection of the v4 and v5 capture regions were 89% (at least 40× coverage), over 80% (at least 50×), and over 60% (at least 100×) (Supplemental Fig. 1, Supplemental Data 1). The percentages of reads mapping to the intersection of these capture regions ranged from 67% to 81% (Supplemental Data 1). Duplication rates ranged from 7% to 25% (Supplemental Data 1).

### Exome-sequencing: somatic copy number analysis

A measure of relative copy number across the genome, log_2_ratio, was obtained by PatternCNV (version 2.02.04). The log_2_ratio was computed by applying the log_2_ transformation to the ratios of the normalized coverage between a tumor sample and a reference set computed for 10 bp windows. The reference set included germline samples from samples that were whole-exome sequenced using the same exome capture methods.

Multi-sample variant calling using GATK’s UnifiedGenotyper^35^ was applied to detect heterozygous variants in the BEAUTY blood samples of these two patients. At each of these sites, we computed the allelic frequencies (AF), the ratio of the number of alternate-allele supporting reads to the total number of reads at the variant site in each of the three tumor samples for the patient in which the heterozygous variant was detected in the blood sample. We performed the transformation 0.5–|0.5–AF| to obtain symmetrically reflected allelic frequencies (RAF). In general, higher values of RAF indicate greater heterozygosity, and lower values indicate less heterozygosity. We evaluated the log_2_ratio and RAF profiles to center the log2ratios to the diploid state.

We segmented the log_2_ratio and RAF profiles using circular binary segmentation implemented in the DNACopy package^36^ with standard-deviation-based segment merging applied at the optimal level as determined by a visual review of the segmentation profiles. We used an in-house method for estimating tumor purity, tumor ploidy, and determining thresholds for the classification of heterozygous variant sites by copy number state. From these classifications, we obtain integrated log2ration/RAF segments. To assess the similarity of copy number alteration profiles across samples by the patient, we calculated empirical probabilities based on copy number at sites of heterozygous variants in the patient blood sample.

### Exome-sequencing: somatic SNV/INDEL analysis

Somatic SNVs were called using SomaticSniper (version 1.0.4.2), JointSNVMix2 (version 0.8b2), and MuTect (version 1.1.7), and somatic indels were called using SomaticIndelDetector (GATK version)^34^ and annotated with variant effect predictor (VEP). Additional criteria were applied to arrive at a filtered set of somatic SNVs and INDELs that were highly likely to be true somatic mutations (Supplemental Data 4). The following filters were applied to call somatic variants: (i) number of variant-supporting reads in the tumor sample must be at least 2, (ii) number of variant-supporting reads in the blood sample must be 0 or must be <1% of the total reads at that site, and (iii) the statistic MQ, the average mapping quality across all reads overlapping the variant site, must be ≥42, (iv) all population frequency estimates reported by VEP must be <0.01, and (v) SNV calls must be made by Mutect or both SomaticSniper and JointSNVMix2. We use the term somatic support to indicate that reads supporting a particular somatic variant were detected in a sample regardless of whether the variant was called in that sample. The presence or absence of somatic SNV/INDEL support provides additional information about somatic variants called in one tumor or tumor-derived sample but not another. We provide Venn diagrams that show overlap for the somatic SNV/INDEL calls and somatic SNV/INDEL support across the three tumor samples for each patient and provided corresponding summary statistics at the sample pair level.

### Exome-sequencing: identification of oncogenic alterations

As part of our characterization of the tumor samples, we have featured several important alterations (Fig. 5). We selected those alterations that were identified as oncogenic or likely oncogenic by the OncoKB resource^37^ and that were also identified as cancer genes by membership in four other cancer gene resources: the MSK-IMPACT clinical test^38^, the FOUNDATION ONE CDx test (Foundation Medicine), Vogelstein et al. (2013)^39^ and the SANGER Cancer Gene Census^40^. For genes identified by these resources, we further used OncoKB^37^, the Cancer Gene Census^40^, and the COSMIC database^41^ to identity those for which the particular types of alterations and, for SNV/INDELs, the particular mutations, are known to be relevant for breast cancer. For loss-of-functions alterations to tumor suppressors, we feature those with inferred loss of function for both alleles. We additionally feature RB1 loss of heterozygosity (LOH) as it has been shown to cause functional loss of RB1 in breast cancer^42^. While CDKN2B was only identified as a cancer gene in 3 of these 5 resources, we also featured it also because the copy number alteration that affected CDKN2A also affected this adjacent cancer gene.

### Reporting summary

Further information on research design is available in the Nature Research Reporting Summary linked to this article.

## Supplementary information

Supplementary Data 1

Supplementary Data 2

Supplementary Data 3

Supplementary Data 4

Supplementary Information

Reporting Summary

## Data Availability

The data generated and analyzed during this study are described in the following data record: 10.6084/m9.figshare.14035853^43^. Whole exome sequencing for each PDX and PDX-derived cell line are openly available in the *Sequence Read Archive* via accession https://identifiers.org/ncbi/insdc.sra:SRP274166^44^ (BioProject accession: PRJNA649579). The following data are openly available as part of the figshare data record^43^: the histology images, in the zip file ‘Fig4_Histology.zip’; additional data supporting the figures and supplementary figures of the related article, in the Excel spreadsheet ‘Extended_Text_and_Tables.xlsx’.

## Materials availability

All materials outlined in this manuscript are available at request by contactingcorresponding author directly.
