# Supplementary material for: Establishment and characterization of immortalized human breast cancer cell lines from breast cancer patient-derived xenografts (PDX)
Source: NPJ Breast Cancer. 2021 Jun 18;7:79. doi: 10.1038/s41523-021-00285-x (PMC8213738; doi:10.1038/s41523-021-00285-x)
Supplement: Supplementary file 5 — Supplementary Information [file 41523_2021_285_MOESM5_ESM.pdf]

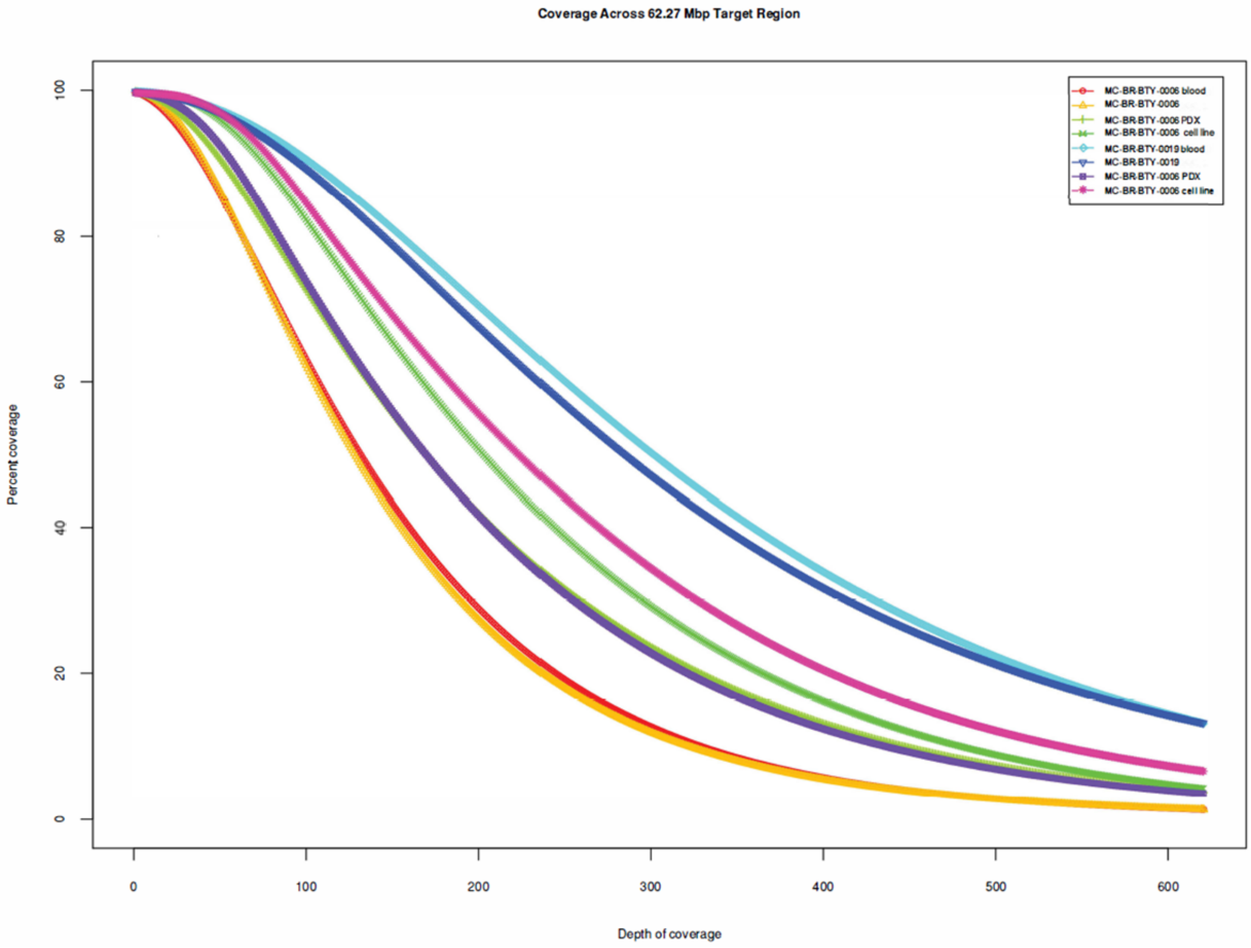

Supplementary Figure 1

For each sample, a coverage profile is produced where a percent of the capture region that is covered is plotted on the vertical axis for each depth of coverage which is plotted on the horizontal axis. Different colors and symbols are used for each sample’s coverage profile as shown in the figure legend. The lowest level of coverage is observed for MC-BR-BTY-0006 blood and MC-BR-BTY-0006 because these samples were sequenced in a ½ lane whereas the others were sequenced in a full lane.

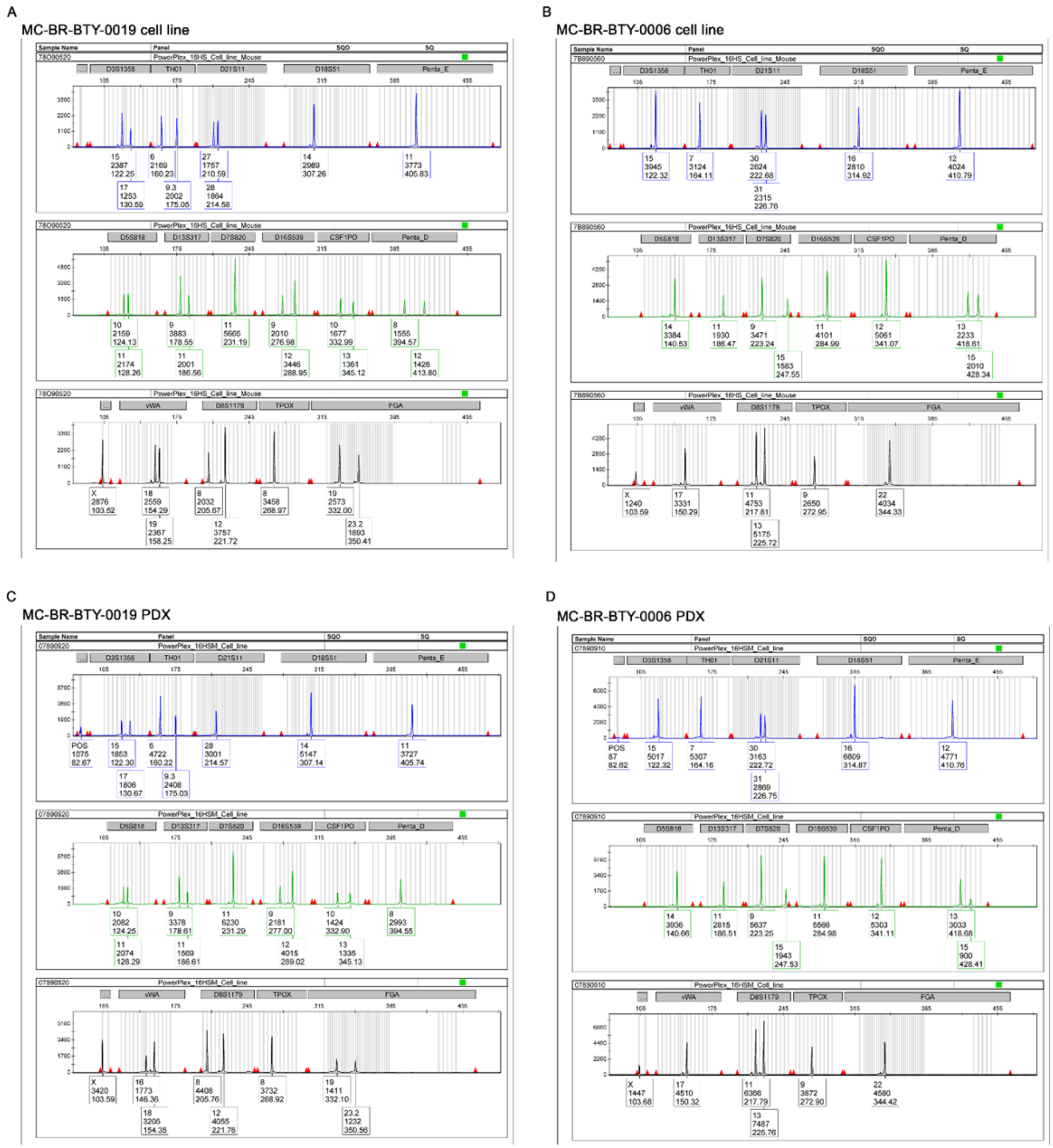

Supplementary Figure 2

Electropherograms of analyzed data for **A.** MC-BR-BTY-0019 cell line and **B.** MC-BR-BTY-0006 cell line and **C.** MC-BR-BTY-0019 PDX and **D.** MC-BR-BTY-0006 PDX. The label inside the grey box is the genetic locus being tested. The red triangles are marks that indicate where that genetic locus' alleles begin and end. The grey bars are the "bins" – these are the place holders for the most common alleles in the human population, alleles that don't fall within the bin would

be considered rare. The numbers in the box below each peak: Top Number: Allele call (STR peak) – this was used to search the reference databases; Middle Number: Peak height (relative fluorescent units (RFUs)); Bottom Number: Sizing (base pairs (bp)) – how far the fragment has traveled in the capillary. The PowerPlex16HS test includes a mouse marker for detection of the presence of mouse DNA, with detection limit as low as 0.5% mouse DNA. If mouse DNA is present, it will be listed as “POS” on the electropherogram.

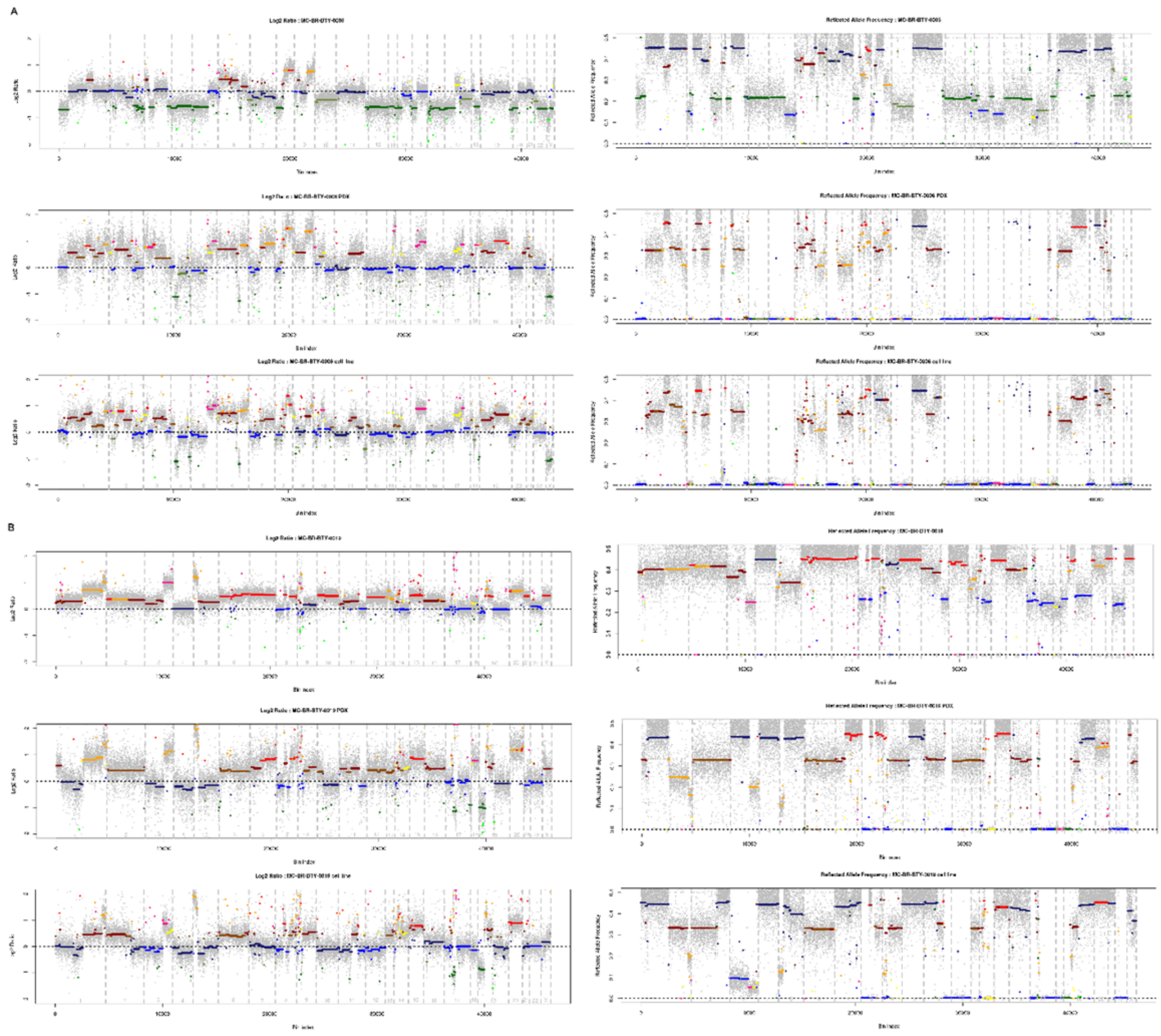

Supplementary Figure 3

The whole genome plots of log_2ratio and Reflected Allele Frequency for **A.** MC-BR-BTY-0019 and **B.** MC-BR-BTY-0006. Dashed vertical lines indicate boundaries between chromosomes. The black dashed lines indicate the estimates diploid state. The gray dots show the observed values at sites called as heterozygous in the blood samples. The combined log_2ratio/RAF segmentation is shown as horizontal lines color-coded by copy number state (**HOMD=green,** **DLOH=dark green**, **DLOH.SC=dark olive green**, **NLOH=blue**, **HET=midnight blue**, **GAIN.SC=dark orange**, **GLOH=yellow**, **GAIN=dark red**, **ALOH=deep pink**, **ASAMP=orange**, **BAMP=red**). These abbreviations for copy number state are defined in Supplemental Table 3.

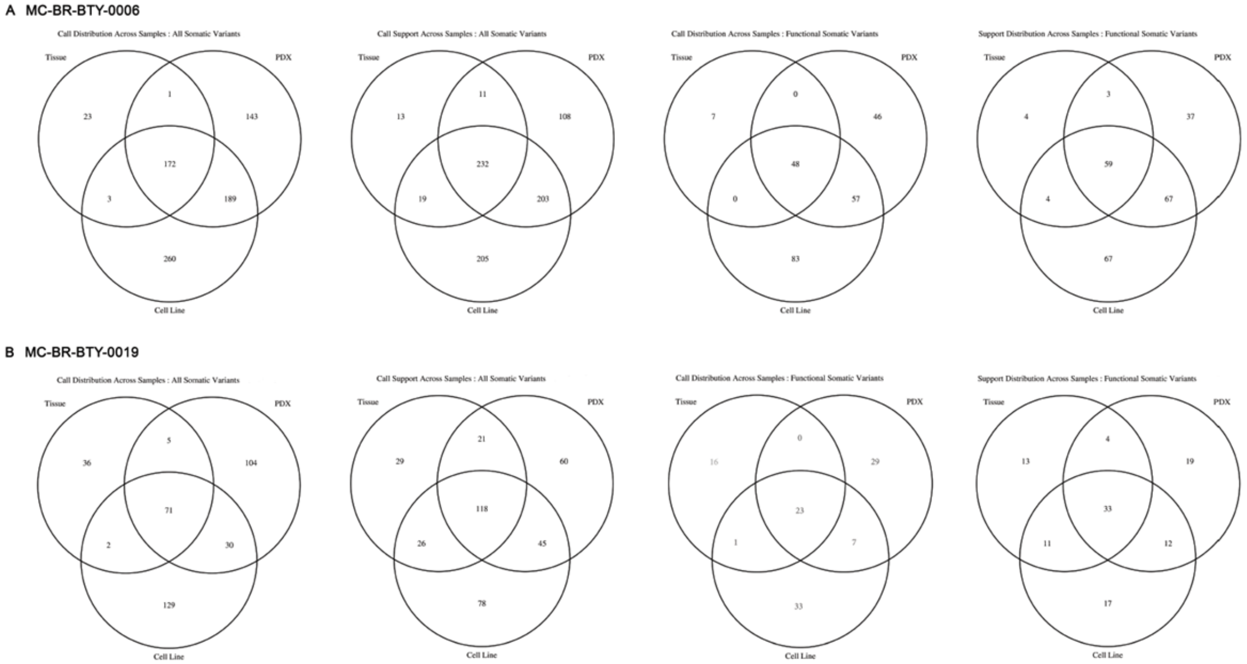

Supplementary Figure 4

Venn diagrams are displayed for somatic SNV/INDELs calls, somatic SNV/INDELs support, and the subset of each of these that are annotated as functional for **A.** MC-BR-BTY-0006 and **B.** MC-BR-BTY-0019. Functional mutations are defined as those that have Variant_Classification, as assigned by VEP, as "Frame_Shift_Del", "Frame_Shift_Ins", "In_Frame_Del","In_Frame_Ins","Missense_Mutation","Nonsense_Mutation","Nonstop_Mutation", "Splice_Site", or "Translation_Start_Site".

A **MC-BR-BTY-0019**

DNA ANALYSIS- 100% MATCH

	D7S820	vWA	D5S818	D13S317	D16S539	TH01	TPOX	CSF1PO	AMEL
MC19 CL 08/2017	11	18, 19	10, 11	9, 11	9, 12	6, 9.3	8	10, 13	X
MC19 CL 02/2019	11	18, 19	10, 11	9, 11	9, 12	6, 9.3	8	10, 13	X

B **MC-BR-BTY-0006**

DNA ANALYSIS- 100% MATCH

	D7S820	vWA	D5S818	D13S317	D16S539	TH01	TPOX	CSF1PO	AMEL
MC06 CL 11/2017	9, 15	17	14	11	11	7	9	12	X
MC06 CL 02/2019	9, 15	17	14	11	11	7	9	12	X

Supplementary Figure 5

STR profile matches for **A.** MC-BR-BTY-0019 cell line and **B.** MC-BR-BTY-0006 cell line at two time points.

RAW Western blot 1

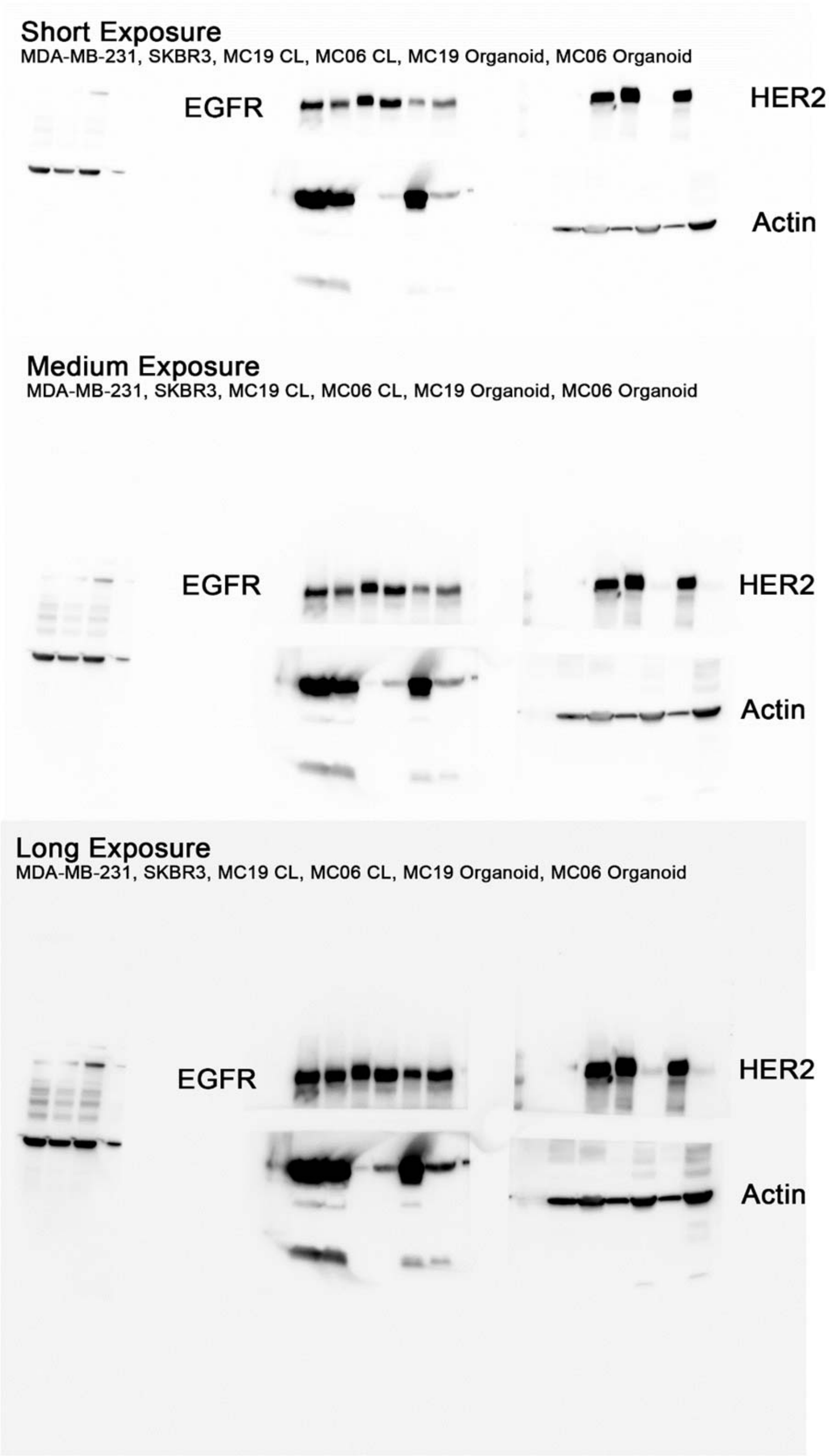

Raw Western blot 2

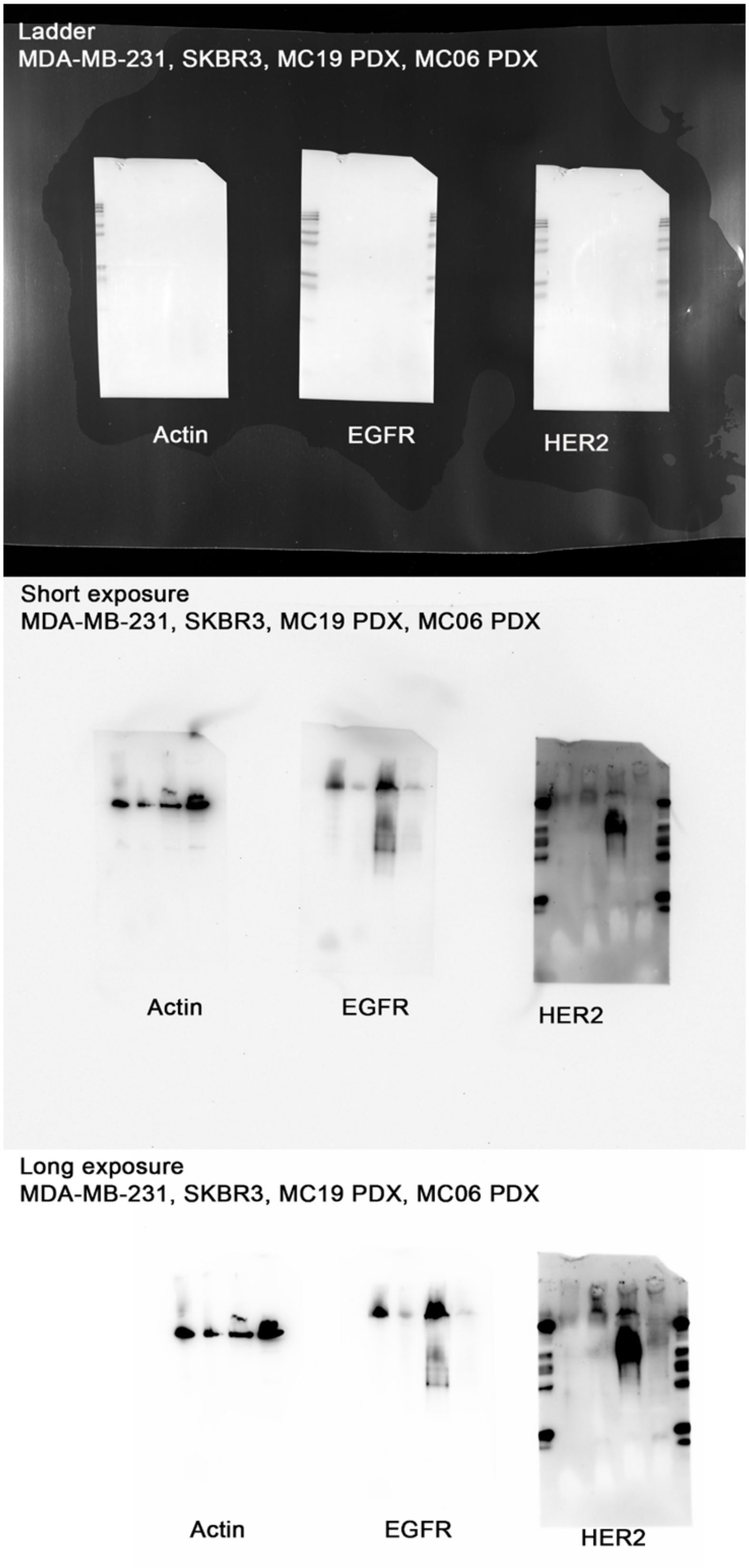

Supplementary Data:

Supplementary Data 1: Alignment and coverage statistics by sample.
Supplementary Data 2: The genomic segmentations by copy number state for each sample. The segments are given in hg19 coordinates.
Supplementary Data 3: Dictionary of copy number states used in Supplemental Figure 3 and Supplemental Table 2.
Supplementary Data 4: Table of the somatic SNV/INDEL variants detected in all samples. The sites are given in hg19 coordinates.
