# Supplementary material for: Establishment and characterization of immortalized human breast cancer cell lines from breast cancer patient-derived xenografts (PDX)
Source: NPJ Breast Cancer. 2021 Jun 18;7:79. doi: 10.1038/s41523-021-00285-x (PMC8213738; doi:10.1038/s41523-021-00285-x)
Supplement: Supplementary file 6 — Reporting Summary [file 41523_2021_285_MOESM6_ESM.pdf]

nature research

Corresponding author(s): LIEWEI WANG

Last updated by author(s): 7/21/2020

Reporting Summary

Nature Research wishes to improve the reproducibility of the work that we publish. This form provides structure for consistency and transparency in reporting. For further information on Nature Research policies, see our Editorial Policies and the Editorial Policy Checklist.

Statistics

For all statistical analyses, confirm that the following items are present in the figure legend, table legend, main text, or Methods section.

n/a	Confirmed	
☐	☒	The exact sample size (n) for each experimental group/condition, given as a discrete number and unit of measurement
☐	☒	A statement on whether measurements were taken from distinct samples or whether the same sample was measured repeatedly
☐	☒	The statistical test(s) used AND whether they are one- or two-sided<br>*Only common tests should be described solely by name; describe more complex techniques in the Methods section.*
☐	☒	A description of all covariates tested
☐	☒	A description of any assumptions or corrections, such as tests of normality and adjustment for multiple comparisons
☐	☒	A full description of the statistical parameters including central tendency (e.g. means) or other basic estimates (e.g. regression coefficient) AND variation (e.g. standard deviation) or associated estimates of uncertainty (e.g. confidence intervals)
☐	☒	For null hypothesis testing, the test statistic (e.g. F, t, r) with confidence intervals, effect sizes, degrees of freedom and P value noted<br>*Give P values as exact values whenever suitable.*
☒	☐	For Bayesian analysis, information on the choice of priors and Markov chain Monte Carlo settings
☒	☐	For hierarchical and complex designs, identification of the appropriate level for tests and full reporting of outcomes
☒	☐	Estimates of effect sizes (e.g. Cohen's d, Pearson's r), indicating how they were calculated

Our web collection on statistics for biologists contains articles on many of the points above.

Software and code

Policy information about availability of computer code

Data collection	GRAPHPAD PRISM Software, HCS v3.3.52 collection software, FASTQC software were utilized
Data analysis	GRAPHPAD PRISM Software, HCS v3.3.52 collection software, FASTQC software were utilized

For manuscripts utilizing custom algorithms or software that are central to the research but not yet described in published literature, software must be made available to editors and reviewers. We strongly encourage code deposition in a community repository (e.g. GitHub). See the Nature Research guidelines for submitting code & software for further information.

Data

Policy information about availability of data

All manuscripts must include a data availability statement. This statement should provide the following information, where applicable:

- Accession codes, unique identifiers, or web links for publicly available datasets
- A list of figures that have associated raw data
- A description of any restrictions on data availability

All data generated or analyzed during this study are included in this published article (and its supplementary information files).

Field-specific reporting

Please select the one below that is the best fit for your research. If you are not sure, read the appropriate sections before making your selection.

☒ Life sciences ☐ Behavioural & social sciences ☐ Ecological, evolutionary & environmental sciences

For a reference copy of the document with all sections, see nature.com/documents/nr-reporting-summary-flat.pdf

Life sciences study design

All studies must disclose on these points even when the disclosure is negative.

Sample size	ALL EXPERIMENTS WERE PERFORMED IN TRIPLICATE
Data exclusions	NO DATA WAS EXCLUDED
Replication	ALL EXPERIMENTS WERE PERFORMED IN TRIPLICATE
Randomization	ALL MICE WERE RANDOMIZED BY STANDARD RANDOMIZATION PROCEDURE WHEN TUMORS ACHIEVED DESIRED SIZE BY RANKING TUMOR VOLUME LARGEST TO SMALLEST AND DIVIDING INTO TWO GROUPS WITH EQUIVALENT AVERAGE TUMOR VOLUME SIZE.
Blinding	MOUSE STUDIES WERE UNBLINDED.

Reporting for specific materials, systems and methods

We require information from authors about some types of materials, experimental systems and methods used in many studies. Here, indicate whether each material, system or method listed is relevant to your study. If you are not sure if a list item applies to your research, read the appropriate section before selecting a response.

Materials & experimental systems

n/a	Involved in the study	
☐	☒	Antibodies
☐	☒	Eukaryotic cell lines
☒	☐	Palaeontology and archaeology
☐	☒	Animals and other organisms
☒	☐	Human research participants
☒	☐	Clinical data
☒	☐	Dual use research of concern

Methods

n/a	Involved in the study	
☒	☐	ChIP-seq
☒	☐	Flow cytometry
☒	☐	MRI-based neuroimaging

Antibodies

Antibodies used	Proteins were detected using the SuperSignal™ West Dura Extended Duration Substrate (BioRad Cat#34075). Primary antibodies against beta-Actin (#4970; 1:1000), HER2/ErbB2 (#2248; 1:200) and EGFR (#4267; 1:1000) were purchased from Cell Signaling. Secondary horseradish peroxidase-linked anti-mouse (#7076s; 1:5000) and anti-rabbit (#7074P2; 1:5000) IgG antibodies were purchased from Cell Signaling.
Validation	ALL ANTIBODIES WERE VALIDATED BY MANUFACTURER

Eukaryotic cell lines

Policy information about cell lines

Cell line source(s)	PDX TUMOR TISSUES
Authentication	STR PROFILING WAS PERFORMED
Mycoplasma contamination	ALL CELL LINES TESTED NEGATIVE FOR MYCOPLASMA
Commonly misidentified lines (See ICLAC register)	*Name any commonly misidentified cell lines used in the study and provide a rationale for their use.*

Animals and other organisms

Policy information about studies involving animals; ARRIVE guidelines recommended for reporting animal research

Laboratory animals	NSG NOD.Cg-Prkdcscid Il2rgtm1Wjl/SzJ (Stock No: 005557) female mice
Wild animals	OUR STUDY DID NOT INVOLVE WILD ANIMALS
Field-collected samples	FIELD SAMPLES WERE NOT COLLECTED
Ethics oversight	MAYO CLINIC IACUC APPROVED ALL MOUSE STUDIES

Note that full information on the approval of the study protocol must also be provided in the manuscript.
